# Factors associated with involuntary admissions: a register-based cross-sectional multicenter study

**DOI:** 10.1186/s12991-020-00323-1

**Published:** 2021-01-07

**Authors:** G. Maina, G. Rosso, C. Carezana, E. Mehanović, F. Risso, V. Villari, L. Gariglio, M. Cardano

**Affiliations:** 1 Department of Neurosciences ‘Rita Levi Montalcini’, University of Turin, Torino, Italy; 2 Psychiatric Unit, San Luigi Gonzaga University Hospital of Orbassano, Torino, Italy; 3 Psychiatric Unit, Martini Hospital, ASL Città Di Torino, Piedmont Region, Torino, Italy; 4 Mental Health Department of Cuneo, Piedmont Region, Italy; 5 Neuroscience and Mental Health Department, AOU Città della Salute e della Scienza, Torino, Italy; 6 Departments of Cultures, Politics and Society, University of Turin, Torino, Italy

**Keywords:** Involuntary admission, Compulsory treatment, Mechanical restraint

## Abstract

**Background:**

Despite the EU recommendations on mental health, involuntary admission has been under researched in Italy for a long time and the overall picture of involuntary admission still appears fragmentary. The aims of this study are to evaluate involuntary admission rates in the Piedmont Region (Italy) and to investigate clinical and service-related variables associated with involuntary admission.

**Methods:**

This is a cross-sectional retrospective multicenter study involving all psychiatric inpatients units of the general hospitals of Piedmont Region. Data on hospitalizations during 2016 were collected by consulting hospital discharge registers. The analyses were performed on two samples: 6018 patients (data analysis was run on first hospitalization during the study period for those with multiple admissions) and 7881 inpatient episodes. The association between involuntary admission and socio-demographic and clinical characteristics was examined through *t*-test for continuous variables, and Pearson’s Chi-square test for categorical variables. Multilevel modeling was applied in logistic regression models with two levels: for the first model center and participants and for the second model center and inpatient episodes.

**Results:**

Of 6018 inpatients, 10.1% were admitted involuntarily at first hospitalization, while the overall compulsory treatment rate was slightly lower (9.1%) in the inpatient episodes sample (*n* = 7881). The involuntary admission rates ranged from 0.8 to 21% among study centers. Involuntary admissions were primarily associated with younger age, diagnosis of schizophrenia or substance use disorders, longer duration of hospital stay, mechanical restraint episodes, and fewer subsequent hospitalizations during the study period.

**Conclusions:**

The rate of involuntary admission in the Piedmont Region was lower than the mean rate across countries worldwide. There were noteworthy differences in rates of involuntary admission among psychiatric units, although no relationship was found with characteristics of the psychiatric wards or of the areas where hospitals are located.

## Background

Involuntary admissions usually involve a minor group of inpatients. However, it is well known both in the literature and among practitioners that these practices impact not only on patients and staff but also their families and the larger social network they belong to, as well as all those involved professionally in their implementation. Although involuntary admission can be lifesaving, allowing patients to have medical and psychiatric care and avoiding harm to themselves or others, compulsory measures can weaken the therapeutic relationship and increase the perceived coercion experienced by patients [1], leading to long-term avoidance of mental health support and increasing risk for further coercion as an inpatient [2, 3].

The findings of a recent meta-analysis that involved 77 studies of 22 countries worldwide showed that 23% of the patients had been admitted involuntarily. Factors associated with involuntary hospitalizations included being male, single marital status, unemployment, receiving welfare benefits, diagnosis of a psychotic disorder or bipolar disorder, previous involuntary hospitalizations, and economic deprivation [4].

Across the EU, total frequencies of involuntary admissions vary remarkably (about 3 to 30% of all inpatient episodes), and this variation may be influenced by the differences in legal frameworks or procedures [5–7]. In European studies, factors such as diagnosis of schizophrenia, psychotic disorder, mental disorders due to medical conditions or substance use disorders, male gender, and immigrant status, have been related to involuntary admission [8, 9].

Despite the EU recommendations on mental health, involuntary admission has been under researched in Italy for a long time. However, an increasing number of studies have appeared in recent years, although with conflicting results. In 2008, results from a survey of all inpatients at 369 psychiatric facilities for adult acute patients in all Italian Regions except Sicily were published: involuntary admissions accounted for 3.8% of all admissions [10]. A few years later, a research conducted on patients hospitalized from 2011 and 2014 in a psychiatric unit of Perugia, in the Umbria Region, revealed that the rate of involuntary admission was 36.5%; the variables related to involuntary admission were psychotic features, suicidal behavior or impulsive behavior, and not being on medication [11]. Lastly, a research involving 21 Mental Health Departments of the Veneto Region found a prevalence rate of involuntary commitment of 5.3%, with higher percentages in densely population areas, while male gender and psychotic disorders significantly increased compulsory treatments, being single decreased it [12].

In light of these findings, the overall picture of involuntary admission still appears fragmentary and new data are needed in order to better evaluate such an invasive and controversial procedure. Particularly, to date no systematic investigation on the use of involuntary admission procedure has been conducted in Piedmont, a north-western Region of Italy. The first aim of this study was to evaluate involuntary admission rates in the Piedmont Region. The second aim was to investigate socio-demographic, clinical and service-related variables associated with involuntary admission.

## Methods

### Study design and procedures

This is a cross-sectional retrospective multicenter study involving all psychiatric inpatients units for acute care, respectively, located in 25 general hospitals of Piedmont, Italy. The study has been promoted by Piedmont Region, General Directorate of Health Care, and supported by SipPieVa (*Società Italiana di Psichiatria—Sezione Regionale Piemonte e Valle d'Aosta*).

The study coordinator center is the Psychiatric Unit of San Luigi Gonzaga University Hospital of Orbassano. The centers involved in this study are located in the city of Torino, in its first belt or in different municipalities of the seven districts of Piedmont Region. Of 25 centers, 23 provided available data. Of those, 17 centers are located in municipalities of Piedmont districts and 6 (including the coordinator center) in the city of Torino or its first belt. Six of the 23 general hospitals are located in cities with more than 50,000 inhabitants. The maximum number of beds per center was 16 and six had 10 or less (4 minimum).

The data on hospitalizations have been collected by consulting hospital discharge registers and clinical charts in each center during 2016. Variables included socio-demographic characteristics of patients, access mode, reasons for hospitalization (diagnostic macro areas), type of hospitalization (voluntary or compulsory), days of hospital stay, mechanical restraint episodes, discharge type, and main diagnosis at discharge according to ICD-9-CM.

Certified psychiatrists or residents in psychiatry supervised by senior psychiatrists performed the data collection and analysis.

For the purpose of this study, the sample was divided according to whether or not the patients underwent involuntary treatment.

The involuntary treatment in Italy is a medical and legal act, established by Reform Law 180 and currently regulated by articles 33–35 of the law n. 833/1978, which occurs when three criteria are met: (1) psychic alterations that require urgent therapeutic interventions; (2) the patient does not want to voluntarily undergo the treatment; and (3) timely and suitable out-of-hospital treatment is impossible.

The Law states that involuntary admissions need to be formally authorized by the Mayor of the Municipality where the episode occurs and can be only undertaken in acute psychiatric wards located in public general hospitals [13].

### Statistical analysis

The analyses were performed on two samples: 6018 patients (data analysis was run on first hospitalization during the study period for those with multiple admissions) and 7881 inpatient episodes.

Descriptive data were summarized as mean and SD for continuous variables and as frequency and percentage for categorical variables.

The association between involuntary admission and socio-demographic and clinical characteristics was examined through t-test for continuous variables, and Pearson’s Chi-square test for categorical variables, with post hoc Bonferroni correction in case of more than two categories. Variables, such as marital status, working status, and access mode, had high rate of missing data (more than 30%), whereas the variable “diagnostic macro areas at entrance” had heterogeneous diagnoses, was not based on specific criteria (e.g., DSM or ICD), and often was not confirmed by a thorough psychiatric history (emergency setting). Therefore, these variables were not considered for the examination in regression models. In order to explore the effect of the association with involuntary admission, age, gender, nationality, mechanical restraint, main diagnosis at discharge, length of hospitalization, and number of hospitalizations were examined through bivariate and multivariate logistic regression models. Co-linearity between variables was checked.

Since the data were collected from 23 different centers in Piedmont Region, multilevel modeling was applied in logistic regression models with two levels: for the first model center and participants and for the second model center and inpatient episodes. Study participants with missing values in at least one variable were automatically excluded from the final model. Due to the missing data, the final models on participants and inpatient episodes were performed on 5382 and 7047 (89.4% of the total sample) observations, respectively. Additionally, the association between studied variables and mechanical restraint was examined both in the subsample of 608 involuntarily admitted patients and in the subsample of 715 involuntary admissions. Odds Ratios (ORs), 95% confidence intervals, and *p*-value < 0.05 were estimated as the measures of association between the studied correlates and the dependent variable.

All analyses were performed using STATA statistical package, version 12.0 (Stata Corporation, 2011, College Station, TX, USA).

## Results

**Fig. 1:**
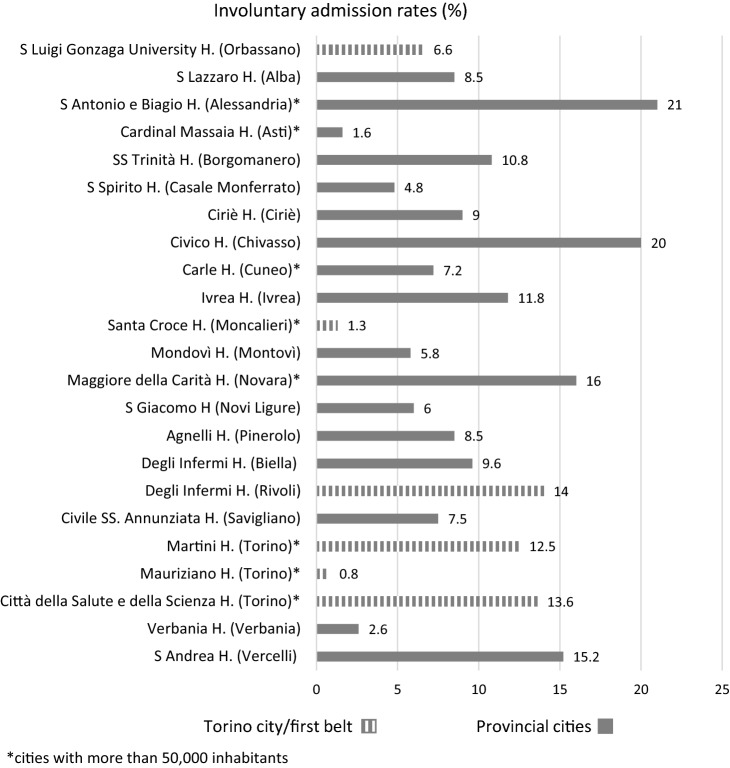
Involuntary admission rates by study center

Of 6,018 inpatients, 10.1% were admitted involuntarily at psychiatric units at first hospitalization. The overall compulsory treatment rate was slightly lower (9.1%) in hospitalization sample (*n* = 7,881). The compulsory treatment rates by study center are shown in Fig. 1: rates ranged from 0.8 to 21%, although no correlation between characteristics of the psychiatric units (number of beds, location in cities with less or more than 50000 inhabitants or in city/first belt vs. more rural areas) and involuntary treatments has emerged.

All socio-demographic characteristics and clinical data of patients are summarized in Tables 1, 2. The mean age of study participants was 46.5 (± 15.7) years on overall, 43.4 (± 15.2) for those admitted involuntarily, while voluntarily admitted patients were older (46.9 ± 15.7). A greater proportion of males than females (11.9% vs 8.2%) and a greater proportion of non-EU and EU citizens versus Italian citizens (16.0% and 12.7% vs 10.2%) were admitted involuntarily at first hospitalization. A higher proportion of involuntary admitted patients were single and unemployed compared to those admitted voluntarily. Regarding clinical variables, a higher proportions of “picked up by ambulance” access mode (44.1% vs 30.9%), psychosis (61.5% vs 33.5%), abnormal behavior (15.9% vs 8.0%) and manic episode (10.1% vs 5.1%) diagnostic macro areas at the entrance, mechanical restraint episodes (26.6% vs 4.6%), and schizophrenic disorders (52.0% vs 29.9%) and substance use disorder (4.9% vs 3.1%) diagnosis at discharge were found in involuntary compared to voluntarily admitted patients. The average number of inpatient days at first hospitalization was 12.2 (± 11.2) on overall, 15.1 (± 11.6) days for involuntarily, and 11.8 (± 11.1) days for voluntarily hospitalized patients (*p* < 0.001).

**Table 1:** Socio-demographic and clinical data: recruited patients sample (n = 6018)

Characteristics	Involuntary admission *n * = 608 *n *(%)	Voluntary admission *n * = 5410 *n *(%)	Overall *n * = 6018 *n *(%)	Stat		
				χ^2^ / *t*	d.f	*p*
Marital status				13.933	3	0.003
Married	87 (25.5)	1024 (33.2)^a^	1111 (32.5)			
Widow	12 (3.5)	135 (4.4)	147 (4.3)			
Divorced	28 (8.2)	316 (10.3)	344 (10.0)			
Single	214 (62.8)^a^	1607 (52.1)	1821 (53.2)			
Educational level				4.132	5	0.634
None	9 (3.5)	85 (3.4)	94 (3.4)			
Primary school	29 (11.3)	275 (10.9)	304 (10.9)			
Secondary school	122 (47.5)	1180 (46.6)	1302 (46.7)			
High school	77 (30.0)	801 (31.7)	878 (31.5)			
University degree	4 (1.6)	36 (1.4)	40 (1.4)			
Major degree	16 (6.1)	153 (6.0)	169 (6.1)			
Working status				15.43	5	0.009
Unemployed	96 (32.0)^a^	734 (26.0)	830 (26.6)			
Employed	84 (28.0)	864 (30.5)	948 (30.3)			
Student	21 (7.0)	155 (5.5)	176 (5.6)			
Housewife	17 (5.7)	237 (8.4)	254 (8.1)			
Retired	28 (9.3)	418 (14.8)^a^	446 (14.3)			
Other (e.g. occasional work)	54 (18.0)	417 (14.8)	471 (15.1)			
Access mode				40.027	5	< 0.001
Picked up by ambulance	214 (44.1)^a^	1307 (30.9)	1521 (32.4)			
Referred to emergency room	239 (49.3)	2603 (61.6)^a^	2842 (60.4)			
Transferred from Intensive Short Observation Unit	1 (0.2)	20 (0.5)	21 (0.4)			
Transferred from other ward (same hospital)	0 (0.0)	21 (0.5)	21 (0.4)			
Transferred from other hospital (public health system)	31 (6.4)	254 (6.0)	285 (6.0)			
Transferred from other health facilities (private)	0 (0.0)	21 (0.5)	21 (0.4)			
Main diagnosis at the entrance (diagnostic macro areas)				222.029	9	< 0.001
Psychosis	220 (61.5)^a^	1128 (33.5)	1348 (36.2)			
Depressive anxious syndrome	15 (4.2)	1040 (30.9)^a^	1055 (28.3)			
Manic episode	36 (10.1)^a^	171 (5.1)	207 (5.6)			
Personality disorder	10 (2.8)	326 (9.7)^a^	336 (9.0)			
Obsessive–compulsive disorder	0 (0.0)	16 (0.5)	16 (0.4)			
Alcohol use disorder	7 (2.0)	87 (2.6)	94 (2.5)			
Substance use disorder	3 (0.8)	36 (1.1)	39 (1.0)			
Suicide attempt	8 (2.2)	259 (7.7)^a^	267 (7.2)			
Abnormal behavior	57 (15.9)^a^	270 (8.0)	327 (8.8)			
Psych. disorders due to medical condition	2 (0.6)	32 (1.0)	34 (0.9)			
Discharge (type)				4.721	4	0.580
At home	473 (78.1)	4120 (76.5)	4593 (76.7)			
Long-term care facility	65 (10.8)	719 (13.4)	784 (13.0)			
Other psychiatric ward (different hospital)	47 (7.8)	405 (7.5)	452 (7.6)			
Therapeutic community	18 (3.0)	118 (2.2)	136 (2.3)			
Other ward of the hospital	2 (0.3)	19 (0.4)	21 (0.4)			
Main diagnosis at discharge (ICD-9-CM codes)				304.885	19	< 0.001
Dementias (290)	10 (1.6)	47 (0.9)	57 (1.0)			
Alcohol-induced mental disorders (291)	3 (0.5)	15 (0.3)	18 (0.3)			
Drug-induced mental disorders (292)	6 (1.0)	32 (0.6)	38 (0.6)			
Schizophrenic disorders (295)	156 (25.7)^a^	931 (17.3)	1087 (18.1)			
Episodic mood disorders (296)	*103 (16.9)*^*a*^	*591 (9.3)*	*604 (0.1)*			
*Manic/mixed episodes*	*1 (0.2)*	*210 (3.9)*^*a*^	*211 (3.5)*			
* Bipolar depression*	*14 (2.3)*	*245 (4.5)*^*a*^	*259 (4.3)*			
* Bipolar disorder NOS*	*6 (1.0)*	*185 (3.4)*^*a*^	*191 (3.2)*			
* Mood disorders NOS*	*9 (1.5)*	*718 (13.3)*^*a*^	*727 (12.1)*			
* Unipolar depression*	37 (6.1)^a^	116 (2.2)	153 (2.6)			
Delusional disorders (297)	123 (20.2)^a^	562 (10.4)	685 (11.4)			
Other nonorganic psychoses (298)	16 (2.6)^a^	58 (1.1)	74 (1.2)			
Pervasive developmental disorders (299)						
Anxiety, dissociative and somatoform disorders (300)	2 (0.3)	132 (2.4)^a^	134 (2.2)			
Personality disorders (301)	65 (10.7)	929 (17.2)^a^	994 (16.6)			
Alcohol dependence (303)	22 (3.6)^a^	91 (1.7)	113 (1.9)			
Substance dependence (304)	3 (0.5) 3 (0.5)	3 (0.5)	20 (0.3)			
Nondependent abuse of drugs (305)	5 (0.8)	57 (1.1)	62 (1.0)			
Acute reaction of stress (308)	6 (1.0)	114 (2.1)	120 (2.0)			
Adjustment reaction (309)	4 (0.7)	103 (1.9)^a^	107 (1.8)			
Others	17 (2.8)	326 (6.0)^a^	343 (5.7)			

^a^Statistically significant (Bonferroni correction)

**Table 2:** Multilevel logistic regression model: variables associated with involuntary admission (patients sample: n = 6018)

Characteristics	Involuntary admission* n* = 608	Voluntary admission* n* = 5410	Overall *n* = 6018	COR (95% CI)^a^	*p*-value	AOR (95% CI)^b^ n = 5382	*p*-value
	n(%)	n(%)	n(%)				
Age (years)							
Mean ± SD	43.4 ± 15.2	46.9 ± 15.7	46.5 ± 15.7	0.99 (0.98–0.99)	< 0.001	0.99 (0.98–0.99)	0.003
Gender							
Female	245 (40.7)	2757 (51.1)	3002 (50.1)	1		1	
Male	357 (59.3)	2638 (48.9)	2995 (49.9)	1.56 (1.31–1.86)	< 0.001	1.19 (0.97–1.45)	0.089
Nationality							
Italian	533 (88.2)	4689 (91.9)	5222 (91.6)	1		1	
EU citizens	24 (4.0)	165 (3.2)	189 (3.3)	1.22 (0.78–1.90)	0.392	0.82 (0.50–1.37)	0.458
Non-EU citizens	47 (7.8)	246 (4.8)	293 (5.1)	1.68 (1.20–2.36)	0.002	1.16 (0.78–1.72)	0.476
Physical restraint							
No	446 (73.4)	5159 (95.4)	5605 (93.1)	1		1	
Yes	162 (26.6)	251 (4.6)	413 (6.9)	14.14 (10.81–18.51)	< 0.001	12.57 (9.35–16.91)	< 0.001
Main diagnosis at discharge							
Mood disorders	133 (21.9)	1859 (34.5)	1992 (33.2)	1		1	
Schizophrenia	316 (52.0)	1609 (29.9)	1925 (32.1)	2.94 (2.36–3.66)	< 0.001	2.65 (2.07–3.38)	< 0.001
Personality disorders	65 (10.7)	929 (17.2)	994 (16.6)	1.02 (0.74–1.39)	0.925	0.92 (0.64–1.31)	0.637
Substance use disorders	30 (4.9)	165 (3.1)	195 (3.2)	2.88 (1.83–4.52)	< 0.001	2.51 (1.53–4.13)	< 0.001
Others^c^	64 (10.5)	827 (15.3)	891 (14.9)	1.07 (0.77–1.48)	0.701	0.92 (0.65–1.31)	0.644
Length of hospitalization (days)							
Mean ± SD	15.1 ± 11.6	11.8 ± 11.1	12.2 ± 11.2	1.02 (1.02–1.03)	< 0.001	1.02 (1.01–1.03)	< 0.001

^a^*COR* Crude Odds Ratios

^b^*AOR* Adjusted Odds Ratios

^c^Dementias; pervasive developmental disorders; anxiety, dissociative and somatoform disorders; adjustment reactions; acute reactions of stress

In the multivariate model (Table 2), male gender was marginally associated with the probability of involuntary admission (OR 1.19, 95% CI 0.97–1.45), while nationality lost significance; the probability of involuntary admission was related with age, with 1% decreased odds for each year of increase in age. Mechanical restraint was associated with 12 times greater odds of involuntary hospitalization compared to patients with no mechanical restraint. The patients with diagnosis of schizophrenia and substance use disorders were associated with 2 times higher probability of involuntary admission compared to patients with mood disorders (OR 2.65, 95% CI 2.07–3.38 for schizophrenia, and OR 2.51, 95% CI 1.53–4.13 for substance use disorders). For each day of increase in hospitalization length, the odds of involuntary treatment increased 1.2-fold (by 2%).

We repeated analysis considering the total number of hospitalization episodes during 2016 (*n* = 7,881). Data on clinical characteristics by psychiatric hospitalizations are shown in Tables 3, 4. All the clinical characteristics are congruent with previous data assessed by admitted patients. In addition, there were a greater proportion of involuntary admissions in those who were admitted once. Even after adjustment, this association remained strong, with 48%, 66%, and 61% lower odds of involuntary treatment for 2, 3, and ≥ 4 hospitalizations, respectively, compared to one hospitalization.

**Table 3:** Clinical variables, hospital/psychiatric ward characteristics, and seasons of the year associated with involuntary or voluntary admissions: inpatient episodes sample (*n* = 7881)

Characteristics	Involuntary admission * n* = 715 * n*(%)	Voluntary admission* n* = 7166 * n*(%)	Overall *n* = 7881 n (%)	Stat		
				χ^2^ / *t*	d.f	*p*
Access mode				41.72	5	< 0.001
Picked up by ambulance	250 (43.4)^a^	1751 (31.1)	2001 (32.2)			
Referred to emergency room	290 (50.3)	3470 (61.6)^a^	3760 (60.5)			
Transferred from Intensive Short Observation Unit	1 (0.2)	34 (0.6)	35 (0.6)			
Transferred from other ward (same hospital)	0 (0.0)	27 (0.5)	27 (0.4)			
Transferred from other hospital (public health system)	34 (5.9)	320 (5.6)	354 (5.8)			
Transferred from other health facilities (private)	1 (0.2)	33 (0.6)	34 (0.5)			
Main diagnosis at the entrance (diagnostic macro areas)				241.048	9	< 0.001
Psychosis	263 (60.3)^a^	1532 (34.7)	1795 (37.0)			
Depressive anxious syndrome	23 (5.3)	1304 (29.5)	1327 (27.4)			
Manic episode	44 (10.1)^a^	223 (5.1)	267 (5.5)			
Personality disorder	8 (1.8)	121 (2.7)	129 (2.7)			
Obsessive–compulsive disorder	4 (0.9)	45 (1.0)	49 (1.0)			
Alcohol use disorder	15 (3.4)	483 (10.9)	497 (10.2)			
Substance use disorder	0 (0.0)	21 (0.5)	21 (0.4)			
Suicide attempt	9 (2.1)	302 (6.8)	311 (6.4)			
Abnormal behavior	68 (15.6)^a^	349 (7.9)	417 (8.6)			
Psych. disorders due to medical condition	2 (0.5)	35 (0.8)	37 (0.8)			
Discharge (type)				6.071	4	0.415
At home	567 (78.2)	5499 (77.2)	6066 (77.3)			
Long-term care facility	80 (11.2)	975 (13.7)	1055 (13.5)			
Other psychiatric ward (different hospital)	54 (7.6)	480 (6.7)	534 (6.8)			
Therapeutic community	19 (2.7)	149 (2.1)	168 (2.1)			
Other ward of the hospital	2 (0.3)	23 (0.3)	25 (0.3)			
Hospital location				3.565	1	0.061
City of Torino/first belt (*n* = 6)	166 (23.2)	1897 (26.5)	2063 (26.2)			
Other cities (n = 17)	549 (76.8)	5269 (73.5)	5818 (73.8)			
Hospital of densely populated areas (> 50,000 inhabitants)				2.663	1	0.108
Yes (*n* = 8)	365 (51.0)	3429 (47.9)	3794 (48.1)			
No (*n* = 15)	350 (49.0)	3737 (52.1)	4087 (51.9)			
Hospital beds per psychiatric ward				1.451	1	0.228
> 10	560 (78.3)	5469 (76.3)	6029 (76.5)			
≤ 10	155 (21.7)	1697 (23.7)	1852 (23.5)			
Seasons of the year				4.271	1	0.234
Winter	177 (24.8)	1814 (25.3)	1991 (25.3)			
Spring	201 (28.1)	1783 (24.9)	1984 (25.2)			
Summer	186 (26.0)	1885 (26.3)	2071 (26.3)			
Fall	151 (21.1)	1678 (23.4)	1829 (23.2)			

^a^Statistically significant (Bonferroni correction)

**Table 4:** Multilevel logistic regression model: variables associated with involuntary admission (inpatient episodes sample: n = 7881)

Characteristics	Involuntary admission n = 715	Voluntary admission n = 7166	Overall n = 7881	COR (95% CI)^1^	*p*-value	AOR (95% CI)^2^ n*n*= 7047	*p*-value
	*n*(%)	*n*(%)	*n*(%)				
Physical restraint							
No	519 (72.6)	6800 (94.9)	7319 (92.9)	1		1	
Yes	196 (27.4)	366 (5.1)	562 (7.1)	12.96 (10.24–16.41)	< 0.001	11.58 (8.90–15.07)	< 0.001
Main diagnosis at discharge							
Mood disorders	148 (20.7)	2375 (33.2)	2523 (32.1)	1		1	
Schizophrenia	375 (52.4)	2226 (31.2)	2601 (33.1)	2.89 (2.35–3.55)	< 0.001	2.68 (2.13–3.36)	< 0.001
Personality disorders	84 (11.8)	1333 (18.7)	1417 (18.0)	1.02 (0.77–1.36)	0.880	0.98 (0.71–1.36)	0.919
Substance use disorders	34 (4.8)	212 (3.0)	246 (3.1)	3.04 (2.00–4.64)	< 0.001	2.60 (1.64–4.14)	< 0.001
Others^c^	74 (10.3)	996 (13.9)	1070 (13.6)	1.11 (0.82–1.51)	0.501	0.94 (0.68–1.31)	0.722
Length of hospitalization (days)							
Mean ± SD	15.8 ± 13.5	12.0 ± 11.3	12.3 ± 11.6	1.03 (1.02–1.04)	< 0.001	1.02 (1.01–1.03)	< 0.001
Number of hospitalizations							
One	608 (85.0)	5410 (75.5)	6018 (76.4)	1		1	
Two	72 (10.1)	1094 (15.3)	1166 (14.8)	0.63 (0.49–0.82)	< 0.001	0.52 (0.39–0.69)	< 0.001
Three	17 (2.4)	366 (5.1)	383 (4.9)	0.46 (0.28–0.76)	0.002	0.34 (0.20–0.58)	< 0.001
Four or more	18 (2.5)	296 (4.1)	314 (4.0)	0.58 (0.36–0.95)	0.032	0.39 (0.23–0.67)	0.001

^a^*COR* Crude Odds Ratios

^b^*AOR* Adjusted Odds Ratios (adjusted for age, gender and nationality)

^c^Dementias; pervasive developmental disorders; anxiety, dissociative and somatoform disorders; adjustment reactions; acute reactions of stress

Finally, the correlates of mechanical restraint (*n* = 162) vs no mechanical restraint (*n* = 446) were examined in the subsample of 608 involuntarily admitted patients. In adjusted model, older age was associated with decreased odds (OR 0.98, 95% CI 0.97–0.99), whereas male gender (OR 3.61, 95% CI 2.14–6.08) and longer length of hospitalization stay (OR 1.03, 95% CI 1.00–1.05) were associated with the increased probability of mechanical restraint. As regard to diagnosis, the probability of mechanical restraint was greater in patients with mood disorders (OR 1.81, 95% CI 1.05–3.14), personality disorders (OR 2.90, 95% CI 1.38–6.09), and substance use disorders (OR 4.02, 95% CI 1.22–13.29). The same analyses were repeated on the total number of involuntary hospitalizations (*n *= 715) and the results were superimposable.

## Discussion

Millions of people experience mental problems in Italy, with about 200,000 hospitalizations each year due to psychiatric disorders. In Piedmont, a north-western Region of the country, approximately 800 admissions per month occur in one of the 25 acute psychiatric wards of the public general hospitals. To date, no systematic data have been collected on involuntary hospitalizations in this Region, although the topic has crucial implications in public health and society.

We found an involuntary admission rate of 10.1% among patients at their first hospitalization during the study period. Even considering the total number of hospitalizations in 2016, the involuntary hospitalization rate was similar (9.1%). This rates are higher than 5.3% found in the Veneto study on the register data of hospital admissions between 2000 and 2007 [12] and much lower than those of other recent studies in different Regions of Italy, where involuntary admission rates reached 36.4% [11] and 40% [14] of overall hospitalizations. This could be due to methodological issues or may be due to Regional differences in terms of approach to psychiatric care, since in Italy the management of public health is demanded to Regions. However, we found an extreme variability of involuntary treatments rate—from 0.8 to 21%—also among the 23 centers of Piedmont Region involved in the present study, although no correlation between involuntary treatments and characteristics of the psychiatric units or of the areas where hospitals are located has emerged. Still, no relationship between seasonality and risk of involuntary admission has been found, contrary to a previous study in 2016 [15]. Based on these findings, it could be argued that differences in involuntary treatment rates mainly depend on unexplored variables, such as Community Mental Health Centers (CMHCs) efficiency in management of critical cases, clinical decision-making processes in emergency room, or even ideological orientation of the psychiatric staff, doctors in particular, toward the formalization of involuntary treatments. It should be highlighted that involuntary admission, psychopharmacological treatments, and psychiatric care itself are harshly criticized by a growing part of the society in Italy.

In bivariate analyses, we found an association between involuntary treatments and socio-demographic features, such as younger age, male gender, occupational status (unemployed), marital status (unmarried), and nationality (non-EU citizens). These results were expected and are in line with previous reports [4, 8, 9, 12]. While younger age and male gender refer to a higher risk of serious and disruptive conditions (e.g., manic episodes, acute episodes of schizophrenia, and substance abuse), unemployment, being single, and non-EU nationality can be considered as indicators of lack of socio-economic support and psychosocial network. However, among socio-demographic variables, the adjusted multilevel logistic regression model confirmed only younger age as factor associated with involuntary admission. To our knowledge no previous studies found a significant association between younger age and involuntary admission. In addition to the aforementioned greater severity of psychopathologies at young age, this relationship might be explained taking into consideration another finding of this study: involuntary admission relates to fewer hospitalization episodes during the study period. This finding is worthy of interest and it is not consistent with the literature. Previous studies found a higher risk of involuntary admission in patients with previous involuntary hospitalizations [4]. In a psychiatric health system territorially organized, patients tend to refer to the same psychiatric ward if they need hospitalization. Hence, after the first hospital stay, they are familiar with mental health workers and ward environment. Good relationships and experiences during first hospitalization could lead to more easily accepting subsequent admissions when necessary. Our finding could be interpreted as a good outcome of psychiatric staff operating in territorial services and psychiatric units of Piedmont Region. Also, it could represent a useful implication of the territorial approach of the Italian psychiatric care.

The regression model confirmed the association between involuntary admission and longer duration of hospital stay, in line with other reports [11]. Moreover, a strong relationship between involuntary admission and mechanical restraint episodes has been found. At least one episode of mechanical restraint concerns 6.9% of the overall sample of patients and 1 out of 4 of those involuntarily hospitalized. This means that the critical conditions that require this procedure are quite frequent in the daily clinical practice of the Piedmont psychiatric wards. Currently, there is no comprehensive literature overview on the beneficial and/or adverse effects of the use of mechanical and pharmacological restraints in the hospital setting [16]. This type of invasive and restrictive procedure is matter of debate among clinicians and there are opposite approaches that depend on both the individual psychiatric unit and the Regional provisions. Many points should be deepened and discussed regarding the risk/benefit ratio of mechanical moderation, and this should be the goal of subsequent studies.

Schizophrenia and substance use disorders were independently related to involuntary admission in our sample. This finding is in line with previous reports as well [4, 9, 12]. However, the association of substance use disorder (main diagnosis) with involuntary admission is noteworthy, given that it is not strictly considered a psychiatric issue in Italy; moreover, hospitalizations in acute psychiatric wards are not expected for that patients by the health system. This finding could be related to the rising number of emergency room visits for patients with acute intoxication of psychostimulants and/or alcohol leading to abnormal behavior and extreme agitation episodes that cannot be managed without compulsory treatments and hospitalization in psychiatric wards. However, in the subsample of involuntarily admitted patients, a greater proportion of patients with the diagnosis of mood disorders and personality disorders were mechanically restrained. So, although patients with schizophrenia are more frequently admitted involuntarily, they are less prone to be mechanically restrained, while subjects with mood and personality disorders, though being less likely to be involuntarily hospitalized, have higher probability to receive mechanical restraint during an involuntary admission. The analyses on specific diagnostic subgroups reveal that manic/mixed episodes, non-organic psychosis, and delusional disorders are also related to involuntary treatment. Looking at “diagnosis at the entrance,” it is surprising how manic episode is underrepresented compared to psychosis (5.5% vs 37% in overall sample; 10.1% vs 60.3% in involuntary admission sample). This could reflect how the diagnosis of bipolar disorder is still underestimated even in the acute emergency settings, probably due to a greater attention given to psychotic symptoms compared to mood alterations, especially when an involuntary treatment has to be certified.

This study should be considered in light of several limitations. First, the cross-sectional design does not allow drawing inferences about causality. Although we can eliminate the risk of reverse bias for some characteristics, such as age, gender, and nationality, the temporal order for other characteristics remains an unsolved problem. Moreover, due to missing data, the number of observations in the final model was reduced. Some variables of interest were not eligible for further examination due to high proportion of missing data (marital status, working status, access mode, and main diagnosis at the entrance). Another main limitation is related to the lack of data on medications at admission. It is well known both in literature and in clinical practice that lack of adherence is a major risk factor for involuntary hospitalization [17]. Further, comorbid psychiatric disorders (secondary diagnoses) and medical conditions were not analyzed. Lastly, as abovementioned, a major limitation of the study is that variables associated with Community Mental Health Centers (CMHCs) efficiency are missing.

The study also has some strength: sample size, dependent variable (type of psychiatric admission) with no missing values, and multilevel modeling applied to control for hierarchical nature of the data.

## Conclusion

In conclusion, we found a rate of involuntary treatment in Piedmont Region lower than the mean rate of involuntary treatment across countries worldwide. Involuntary admissions are primarily associated with younger age, diagnosis of schizophrenia or substance use disorders, and fewer hospitalizations during the study period. There are noteworthy differences in rates of involuntary admissions among psychiatric units, although no relationship was found with characteristics of the psychiatric wards or of the areas where hospitals are located.

Our findings indicate that involuntary treatment is not applied uniformly in psychiatric units of Piedmont Region and reasons should be deepened through a debate with heads and clinicians of the psychiatric departments. The point is crucial in terms of clinical and social implications and psychiatrists should take a shared position and improve communication to the society on such critical topic.

## Data Availability

The data that support the findings of this study are not openly available, due to confidentiality of data collected from hospital discharge registers and clinical charts.

## Abbreviations

SipPieVa: *Società Italiana di Psichiatria—Sezione Regionale Piemonte e Valle d'Aosta*
ORs: Odds ratios
